# The fatty acid synthase inhibitor triclosan: repurposing an anti-microbial agent for targeting prostate cancer

**DOI:** 10.18632/oncotarget.2433

**Published:** 2014-09-03

**Authors:** Martin C. Sadowski, Rebecca H. Pouwer, Jennifer H. Gunter, Amy A. Lubik, Ronald J. Quinn, Colleen C. Nelson

**Affiliations:** ^1^ Australian Prostate Cancer Research Centre - Queensland, Institute of Health and Biomedical Innovation, Queensland University of Technology, Princess Alexandra Hospital, Translational Research Institute, Brisbane, Australia; ^2^ Eskitis Institute for Drug Discovery, Griffith University, Brisbane, Australia; ^3^ Vancouver Prostate Centre, Department of Urologic Sciences, University of British Columbia, Vancouver, Canada

**Keywords:** Triclosan, fatty acid synthase, AMPK, lipid metabolism, C75, orlistat, prostate cancer

## Abstract

Inhibition of FASN has emerged as a promising therapeutic target in cancer, and numerous inhibitors have been investigated. However, severe pharmacological limitations have challenged their clinical testing. The synthetic FASN inhibitor triclosan, which was initially developed as a topical antibacterial agent, is merely affected by these pharmacological limitations. Yet, little is known about its mechanism in inhibiting the growth of cancer cells. Here we compared the cellular and molecular effects of triclosan in a panel of eight malignant and non-malignant prostate cell lines to the well-known FASN inhibitors C75 and orlistat, which target different partial catalytic activities of FASN. Triclosan displayed a superior cytotoxic profile with a several-fold lower IC50 than C75 or orlistat. Structure-function analysis revealed that alcohol functionality of the parent phenol is critical for inhibitory action. Rescue experiments confirmed that end product starvation was a major cause of cytotoxicity. Importantly, triclosan, C75 and orlistat induced distinct changes to morphology, cell cycle, lipid content and the expression of key enzymes of lipid metabolism, demonstrating that inhibition of different partial catalytic activities of FASN activates different metabolic pathways. These finding combined with its well-documented pharmacological safety profile make triclosan a promising drug candidate for the treatment of prostate cancer.

## INTRODUCTION

Fatty acid synthase (FASN) is a key metabolic enzyme that catalyzes in a stepwise and sequential manner the *de novo* synthesis of fatty acids (FA), predominantly palmitate, from the condensation of seven molecules of malonyl-CoA and one molecule of acetyl-CoA. This NADPH-dependent process plays a central role in energy homeostasis by converting excess carbon intake into FAs for storage [1]. As a homodimeric, multifunctional enzyme, FASN employs seven catalytic activities (β-ketoacyl synthase, malonyl/acetyl transferase, dehydrase, enoyl reductase, β-ketoacyl reductase, and acyl carrier protein) during each cycle of FA chain elongation before its thioesterase activity releases the ultimate product, free palmitate [2].

FASN is expressed at relatively low levels in normal cells (except liver, brain, lung and adipose tissue), whereas it is highly expressed in a wide variety of cancers, including cancer of the prostate, breast, brain, lung, ovary, endometrium, colon, thyroid, bladder, kidney, liver, pancreas, stomach, oesophagus, eye, mesothelium and skin (reviewed in [3]). Elevated expression of FASN has been found in the earliest stages of cancer development and becomes more pronounced during tumor progression. In prostate cancer (PCa), elevated levels of FASN have been linked to poor prognosis, reduced disease-free survival, aggressiveness of disease, and increased risk of death (reviewed in [3]). Despite the presence of high levels of circulating dietary FAs, FASN plays a central role in tumor cell development and survival. Knockdown or pharmacological inhibition of FASN selectively induces cell death of cancer cells and a reduction in tumor volume in xenograft mouse models with only a minimal effect on normal cells, indicating that FASN is a promising target for cancer treatment with the potential for a large therapeutic index (reviewed in [4]).

Several natural and synthetic FASN inhibitors such as the antifungal agent cerulenin and its synthetic derivative C75, the green tea polyphenol epigallocatechin-3-gallate (EGCG) and other flavonoids (luteolin, quercetin, and kaempferol), the β-lactone orlistat as well as the bactericide triclosan have been shown to inhibit cancer cell growth by inducing cell death (reviewed in [4]). Some of these inhibitors have been shown to work by directly binding and inhibiting different active sites of FASN. For example, cerulenin and C75 interact with the β-ketoacyl synthase domain and irreversibly inhibit the condensation reaction (reviewed in [4]). In addition, C75 was found to also inactivate the enoyl reductase and thioesterase partial activities of FASN [5]. EGCG acts through competitive binding inhibition of NADPH and irreversible inactivation of the β-ketoacyl reductase activity [6], orlistat inhibits FASN through formation of a covalent adduct with the thioesterase domain [7], and triclosan (TCS) binds and inactivates the enoyl reductase domain [8]. Given the multi-domain structure of FASN, it is not surprising that the cytotoxic effect of various FASN inhibitors can have different underlying mechanisms, such as end product starvation through depletion of palmitate, or toxic accumulation of the FASN substrate malonyl-CoA or intermediates of FA synthesis.

Although FASN inhibitors showed promising anti-cancer activities, their evaluation in clinical trials was challenged due to pharmacological limitations. Cerulenin was found to be chemically unstable and undesirable for use *in vivo* due to its very reactive epoxy group. This led to the development of the chemically more stable, synthetic derivative C75 [9]. However, studies in mice revealed that C75 and cerulenin cause appetite suppression and profound weight loss through direct activation of carnitine palmitoyltransferase (CPT-1), which leads to increased FA β-oxidation [10]. These concerns have been addressed with the development of C93, a derivative of C75 that does not activate CPT-1 [11]. EGCG as a clinical FASN inhibitor is challenged by its low potency, bioavailability, serum stability and specificity, which is due to its off-target effects (inhibition of several kinases and topoisomerases) (reviewed in [12]). A clinical application of orlistat will require novel formulations, because it is poorly soluble and has an extremely low oral bioavailability [13]. TCS is an FDA-approved topical broad-spectrum antibiotic that inhibits type II enoyl reductase in bacteria [14] and has been in use for more than 30 years in personal hygiene products. TCS strongly binds to bacterial type II enoyl reductases with affinities in the low picomolar range [15]. Although bacterial and human FASN share very little sequence homology, TCS was found a decade ago to also inhibit the enoyl reductase partial activity of human FASN and to block growth of the breast cancer cell lines MCF-7 and SKBr-3 at concentrations in the low micromolar range [8]. Since then, TCS-induced cytotoxicity in cancer cells has been reported for the retinoblastoma Y79 cell line, the epithelial carcinoma KB cell line [16] and the choriocarcinoma JEG-3 cell line [17], whereas similar concentrations of TCS were not cytotoxic to non-malignant cell lines derived from fibroblasts (3T3) or Müller glia (MIOM1) [16, 18]. Moreover, it has been demonstrated in a rat model of mammary carcinogenesis that TCS-mediated inhibition of FASN significantly reduced tumor incidence and tumor numbers per animal, with only minor effects on body weight and no effects on food intake [19]. Similarly, treatment of male rats for 60 days with a daily dose between 5-20 mg/kg TCS did not induce significant changes in body weight at any of the test doses [20]. The lack of any significant weight loss strongly suggests that TCS does not share the side-effects of appetite suppression, CPT-1 activation, and increased FA β-oxidation that impeded the development of cerulenin and C75 into clinical FASN inhibitors.

The fact that PCa is the most frequently diagnosed malignancy in men and the second leading cause of male cancer death in industrialized countries [21] highlights the need for alternative therapeutic strategies to manage this devastating disease. Our interest to evaluate the FASN inhibitor TCS as a potential chemotherapeutic for the treatment of PCa was supported by previous findings that TCS showed minimal toxicity in rats, dogs and baboons when treated daily over several months [22] and that knockdown or pharmacological inhibition of FASN with C75 induced cell death of PCa cells *in vitro* and a reduction in tumor volume *in vivo* (reviewed in [4]). Here, we tested TCS for the first time in various PCa cell lines and compared its cytotoxic potency and phenotypic effects on cell morphology, cell cycle, cellular lipid levels, and expression and regulation of key enzymes involved in energy homeostasis and FA synthesis to the well-known FASN inhibitors C75 and orlistat.

## RESULTS

### Triclosan is cytotoxic in prostate cancer cells

**Figure 1:**
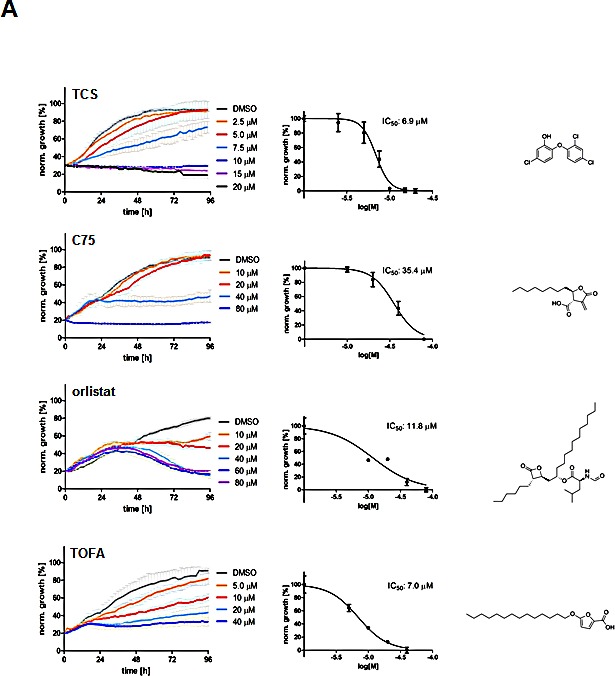

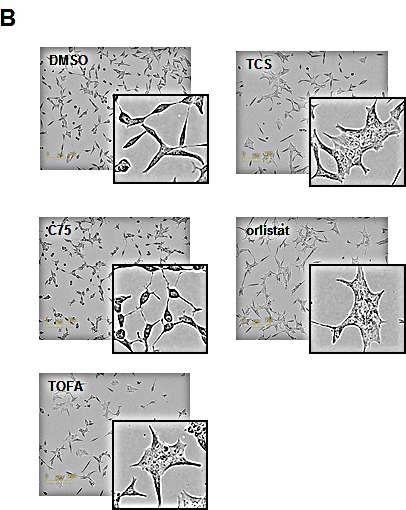

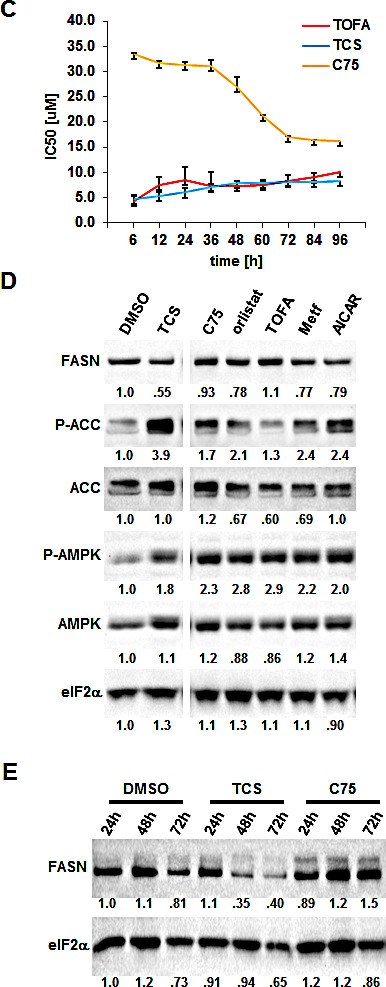

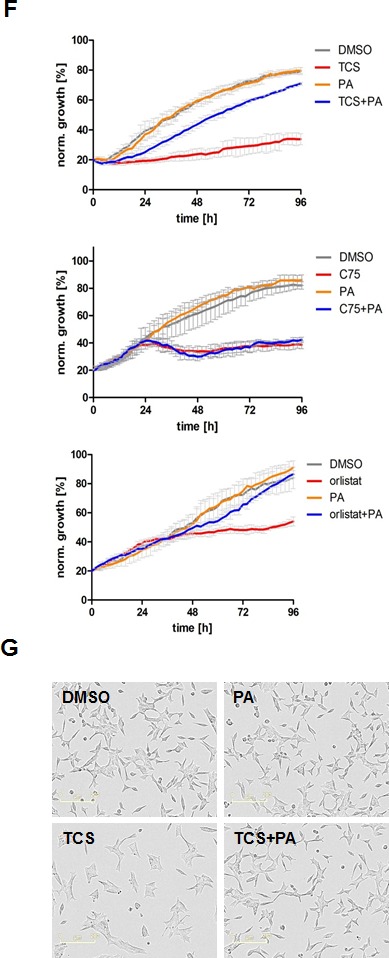
Inhibition of FASN by TCS causes cytotoxicity in PCa cells (A) Proliferation as a function of cell confluence. LNCaP cells were treated with control (DMSO) or the indicated concentrations of inhibitors of *de novo* FA synthesis, and confluence was measured every 2 h for 96 h on an IncuCyte FLR system (left panel). IC50 values for treatment of LNCaP cells for 48 h were calculated by non-linear regression analysis (n=3 ±SD, middle panel). The structures of the lipogenic inhibitors are shown (right panel). (B) Representative images of A after 24 h of treatment with control (DMSO) or inhibitors (TCS 7.5 μM, C75 20 μM, orlistat 30 μM and TOFA 20 μM). (C) IC50 values for the indicated time points were calculated from the data in A (n=3 ±SD). (D) Western blot analysis of key lipogenic and energy sensing enzymes. LNCaP cells were treated for 24 h with control (DMSO) or the indicated inhibitors (TCS 10 μM, C75 20 μM, orlistat 20 μM and TOFA 10 μM), and protein lysates were probed with antibodies directed against the indicated proteins. As controls, LNCaP cells were treated with the AMPK activators metformin (2 mM) and AICAR (0.5 mM). For quantification, total protein levels were normalized relative to loading control (eIF2α). The level of protein phosphorylation was calculated relative to the normalized total amount of the respective protein. For better clarity, irrelevant lanes were removed from the image as indicated by the gaps. (E) LNCaP cells were treated with control (DMSO), 10 μM TCS or 20 μM C75 for the indicated times, and FASN expression was analyzed as in D. (F) Cytotoxicity of TCS and orlistat is mediated by FA starvation. LNCaP cells were treated with control (DMSO), 7.5 μM TCS, 40 μM C75, 10 μM orlistat, 5 μM palmitate (PA), or a combination of FASN inhibitor with PA (TCS+PA, C75+PA and orlistat+PA), and proliferation was measured for 96 h as described in A. (G) Representative images of F after 24 h of incubation.

In order to evaluate the effect of the FASN inhibitor triclosan (TCS) on the growth of the PCa cell line LNCaP and to compare its activity to other FASN inhibitors, we treated cells with TCS (2.5-20 M), C75 (5-50 μM) or orlistat (10-80 μM) and measured cell confluence by live imaging for 96 hours (Fig. 1A). In addition, we included TOFA (5-40 μM) in this analysis, which is an inhibitor of acetyl-CoA carboxylase (ACC), the rate-limiting enzyme of *de novo* FA synthesis which converts acetyl-CoA into malonyl-CoA [23]. As shown in Figure 1A, all inhibitors were cytotoxic to LNCaP cells, and cell death was preceded by loss of cell-cell contacts, cell shrinkage, and membrane blebbing which are typical signs of apoptosis (data not shown). Yet, the timing of growth inhibition and cell death was different between the inhibitors, as indicated by their distinct growth profiles. While LNCaP cells treated with 40 μM of C75, orlistat or TOFA continued to grow between 20-36 h at a similar rate as control before their growth was negatively impacted by the inhibitors, cells treated with 10 μM TCS showed an immediate growth inhibitory effect. Kinetic analysis revealed an IC50 of 6.9 μM for TCS, 35.4 μM for C75, 23.5 μM for orlistat, and 7.5 M for TOFA (Table 1) after treatment of LNCaP cells for 48 h. Measurement of metabolic activity / viability of LNCaP cells treated for 48 h by Alamar Blue assay revealed similar IC50 values (Table 1). Inspection of the images from the time-lapse microscopy (Fig. 1A) revealed that TCS induced cell morphology changes in LNCaP cells after 24 h which were similar to cells treated with orlistat or TOFA, where cells were flat and enlarged and highly granular. This phenotype was very similar to the morphological changes induced by TCS in MCF-7 breast cancer cells [8]. In contrast, C75 caused cells to round up with shortened processes.

**Table:** IC50 in LNCaP cells measured by IncuCyte and Alamar Blue

	IncuCyte			Alamar Blue	
compound	IC50 [μM]	95% CI [μM]		IC50 [μM]	95% CI [μM]
TCS	6.9	6.4-7.4		6.6	6.3-6.9
C75	35.4	32.1-39.1		43.6	39.0-48.6
orlistat	11.8	8.0-17.4		26.4	23.2-30.0
TOFA	7.0	5.7-8.6		13.8	10.4-18.4

Analysis of the cell confluence-derived IC50 values at different time points revealed that TCS and TOFA reached maximum potency in growth inhibition as early as 6 h post treatment, which remained relatively unchanged over the 96 h incubation period (Fig. 1C). In contrast, C75 reached maximum potency (IC50=16.9 μM) after 72 h of incubation, a two-fold increase relative to the IC50 at 6 h (33.6 μM). A similar time-dependent increase of potency was also observed with orlistat (data not shown).

FASN inhibition by TCS was also cytotoxic in the PCa cell lines C4-2B (a castrate-resistent LNCaP derivative), LAPC4, 22RV1 and the metastatic PCa cell line PC-3 (Table 2). While TCS possessed similar IC50 values (4.5 μM to 7.8 μM) in the different PCa cell lines, the potency of C75 varied by more than four-fold between cell lines (8.3 μM to 35.4 μM). Interestingly, the non-malignant prostate cell lines RWPE-1, BPH-1 and WPMY-1 also displayed increased sensitivity to TCS and C75 (Table 2) and orlistat and TOFA (data not shown) when compared to non-malignant 3T3 fibroblasts. Notably, FASN is mainly expressed in adult hormone-sensitive cells like prostate cells or in cells with high lipid metabolism [24]. Consistent with our findings, concentrations of up to 100 μM TCS or C75 showed no cytotoxic effects in the non-malignant fibroblast cell line 3T3-L1 [18].

Previous work has shown that pharmacological inhibition of FASN with C93, a C75 derivative, activates AMP-activated protein kinase (AMPK) in SKOV3 human ovarian cancer cells [11]. AMPK acts as a metabolic master switch with a critical role in sensing cellular energy levels and metabolic stress stimuli (reviewed in [25]). AMPK activation through phosphorylation inhibits *de novo* FA biosynthesis through phosphorylation and repression of the lipogenic transcription factor, SREBP1, which regulates the expression of FASN [11], androgen receptor expression and PCa progression [26, 27]. In addition, activated AMPK directly phosphorylates ACC, thereby inhibiting malonyl-CoA synthesis, the rate-limiting step of *de novo* FA synthesis. Reduced malonyl-CoA levels in turn activate mitochondrial FA β-oxidation through relieved inhibition of carnitine O-palmitoyltransferase 1 (CPT-1) [28]. Hence, we investigated the effects of the lipogenic inhibitors TCS, C75, orlistat and TOFA on protein expression and phosphorylation levels of FASN, ACC and AMPK by Western blot analysis (Figs. 1D and 1E). As positive controls, we treated LNCaP cells with metformin or 5-aminoimidazole-4-carboxamide 1-β-D-ribofuranoside (AICAR) which lead to phosphorylation and activation of AMPK (reviewed in [25]). As shown in Figure 1D, the four lipogenic inhibitors TCS (1.8-fold), C75 (2.3-fold), orlistat (2.8-fold) and TOFA (2.9-fold) caused an increase in AMPK phosphorylation which was similar to metformin (2.2-fold) and AICAR (2.0-fold). Consistent with increased AMPK activity, phosphorylation of ACC (P-ACC) was increased by all three FASN inhibitors. Interestingly, like metformin and AICAR, TCS and orlistat decreased FASN protein levels. Yet, FASN expression remained largely unchanged by C75 and TOFA. Western Blot analysis of a time course experiment revealed that TCS caused a decrease in FASN protein by 2.5-fold after 72 h, while C75 increased FASN protein by 1.5-fold (Fig. 1E). Taken together, inhibition of *de novo* lipogenesis through pharmacological targeting of ACC or FASN increased the phosphorylation of AMPK and its target ACC. Importantly, the FASN inhibitors TCS, C75 and orlistat, which target different partial catalytic activities of FASN, had distinct effects on its protein levels.

**Table:** IC50 in malignant and non-malignant cell lines measured by IncuCyte and Alamar Blue

	TCS			C75	
Cell line	IC50 [μM]	95% CI [μM]		IC50 [μM]	95% CI [μM]
LNCaP	6.9	6.4-7.4		35.4	32.1-39.1
C4-2B	5.9	5.4-6.0		32.1	29.9-34.4
LAPC4	7.8	5.3-11.5		28.3	23.2-32.6
22RV1	4.5	4.0-5.2		9.1	7.8-10.6
PC-3	6.8	6.2-7.4		8.3	7.9-8.8
RWPE-1	0.74	0.66-0.76		1.6	1.4-1.8
BPH-1	10.3	9.6-11.0		15.2	14.0-16.5
WPMY-1	12.9	12.2-13.5		28.2	27.4-29.0
NIH3T3	62.3	58.8-66.1		60.0	56.4-63.8

Next we investigated whether the cytotoxic effect of TCS, C75 and orlistat was caused through end product starvation of palmitate. We performed a rescue experiment where we supplemented TCS-treated LNCaP cells with exogenous palmitate and measured growth in real time (Fig. 1F). When compared to control, addition of palmitate did not affect growth of LNCaP cells. However, supplementation with exogenous palmitate rescued the profound growth inhibition produced by TCS (Fig. 1F). Similarly, exogenous PA recued the growth inhibition induced by orlistat (Fig. 1F), which has been reported previously in PC-3 cells [29]. Furthermore, TCS-treated LNCaP cells did not display the above described morphological phenotype (flat, enlarged and highly granular cells) when co-treated with exogenous palmitate (Fig. 1G). These results indicated that the anti-proliferative and morphological effects of TCS were a result of end product starvation which was mediated through FASN inhibition. In contrast, C75-induced growth inhibition of LNCaP cells could not be rescued by palmitate (Fig. 1F). This observation is consistent with a previous study [30], which suggested that increased malonyl-CoA levels might be the major cause for cytotoxicity of C75 [31]. Altogether, these findings demonstrate that the FASN inhibitor TCS is a potent cytotoxic compound in PCa cells. Furthermore, these results highlight that inhibition of different partial catalytic activities of FASN with different inhibitors can produce distinct phenotypes regarding cell morphology, expression levels of FASN and mechanism of growth inhibition.

### Triclosan, C75 and orlistat have different effects on the expression of lipogenic genes

**Figure 2:**
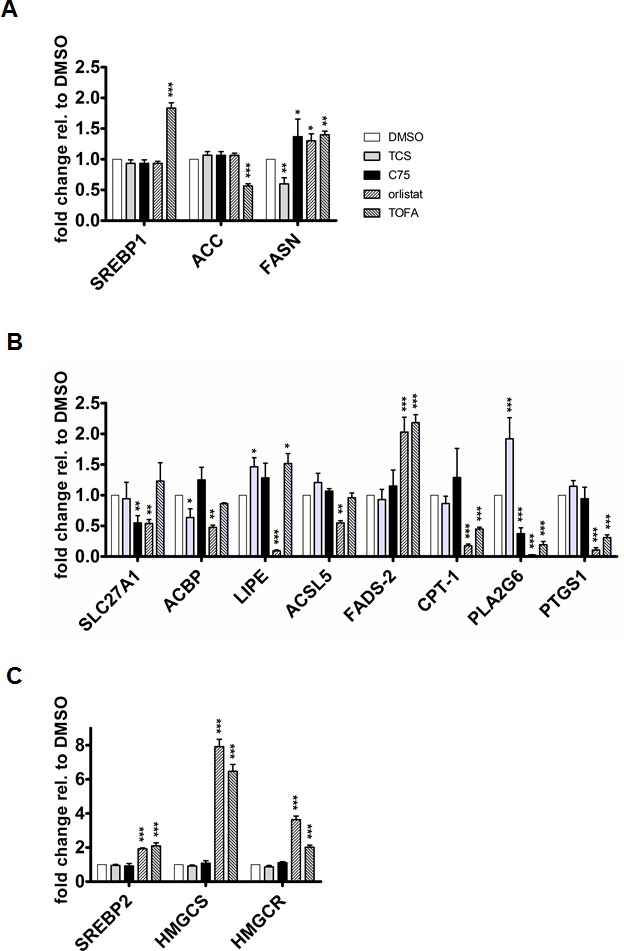
Inhibition of FA synthesis causes distinct effects on the expression of key genes involved in FA and cholesterol metabolism (A) The expression of SREBP1, ACC and FASN was analyzed by qRT-PCR after treatment of LNCaP cells with TCS (5 μM), C75 (20 μM), orlistat (20 μM), and TOFA (10 μM) for 48 h (n=3, mean±SD, *p<0.05, **p<0.01, ***<0.001). (B) The indicated genes involved in various aspects of FA metabolism were analyzed as in A. (C) Effect of FA synthesis inhbitiors on the expression of key enzymes of cholesterol synthesis. SREBP2, HMGCS and HMGCR expression was analyzed as in A.

The above observed changes to FASN protein levels by the inihibitors raised the possibility that these effects were caused through transcriptional changes via SREBP1. In addition, we investigated key genes of FA synthesis (ACC and FASN) and FA metabolism like FA uptake and transport (SLC27A1 and ACBP), FA conversion (HSL/LIPE, ACSL5 and FADS-2), FA β-oxidation (CPT-1), and arachidonic acid metabolism (PLA2G6 and PTGS1) by quantitative real-time PCR (Figs. 2A- 2C). As shown in Figure 2A, TCS, C75 and orlistat did not affect the mRNA expression of SREBP1 and ACC in LNCaP cells (Fig. 2A). In contrast, TOFA led to a significant upregulation of SREBP1 mRNA levels and decrease in ACC expression (Fig 2A). More importantly, TCS significantly reduced the gene expression of FASN, whereas C75, orlistat and TOFA caused a significant increase (Fig. 2A). In addition, the lipogenic inhibitors displayed distinct effects on the transcript levels of FA transporters SLC27A1 and ACBP, hormone-sensitive lipase HSL/LIPE which hydrolyzes stored triglycerides to free FAs, acyl-CoA synthase ACSL5, and FA desaturase FADS-2 (Fig. 2B). We observed that both orlistat and TOFA significantly decreased the expression of carnitine palmitoyltransferase (CPT-1), which controls the rate-limiting step of FA β-oxidation. Of note was also the opposite regulation of phospholipase PLA2G6 expression by TCS compared to C75, orlistat and TOFA. PLA2G6 has been shown to play a role in the release of FA and arachidonic acid from phospholipids [32]. Consistent with a potential effect of orlistat and TOFA on the arachidonic acid pathway, both inhibitors caused a strong decrease in the expression of PTGS1, which catalyzes the conversion of arachidonic acid into prostanglandin [33]. Acetyl-CoA, the building block of FA synthesis, is also a substrate of the mevalonate pathway, which is important for the synthesis of cholesterol, sterols and other isoprenoids. The FA and cholesterol synthesis pathways are coordinately regulated by a feedback system mediated by SREBP1 and SREBP2 [34]. SREBP2 regulates the expression of hydroxy-methyl-glutaryl-CoA synthase (HMGCS) and hydroxy-methyl-glutaryl-CoA reductase (HMGCR), two key enzymes of cholesterol synthesis. In addition, previous work has shown that C75 and TOFA inhibited sterol synthesis in primary hepatocytes [35]. Hence, we tested if FA synthesis inhibition affected the expression of SREBP2, HMGCS, and HMGCR by qRT-PCR (Fig. 2C). In contrast to TCS and C75, orlistat and TOFA caused a significant up-regulation in the expression of SREBP2, HMGCS and HMGCR, suggesting that the latter two inhbitors stimulate cholesterol synthesis in LNCaP cells through a feedback mechanism. In summary, inhibition of *de novo* FA synthesis with different ACC and FASN inhibitors activates distinct transcriptional responses of genes involved FA and cholesterol metabolism.

### Triclosan causes a G0/G1 cell cycle arrest and induces apoptosis in PCa cells

**Figure 3:**
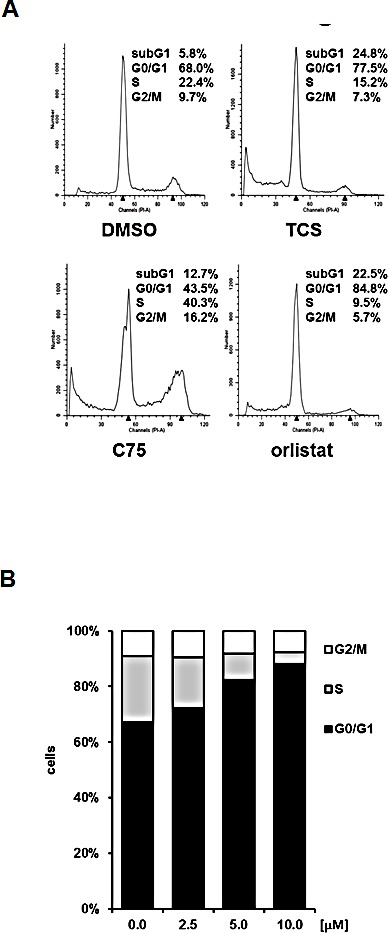

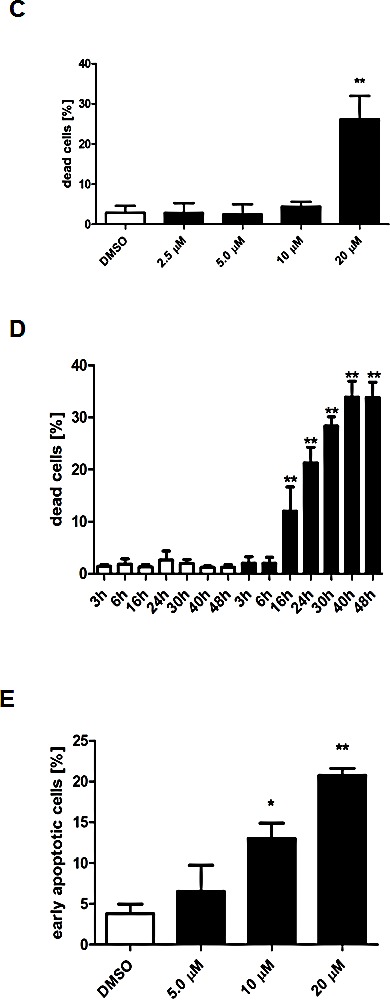

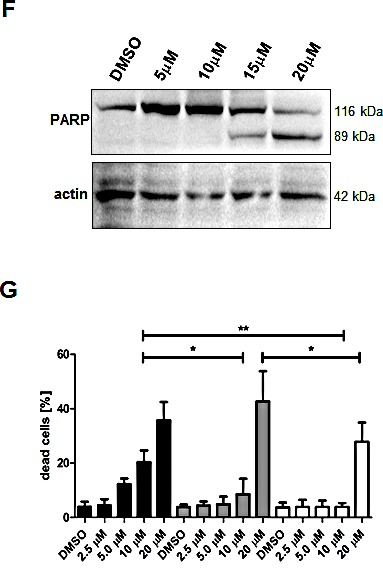

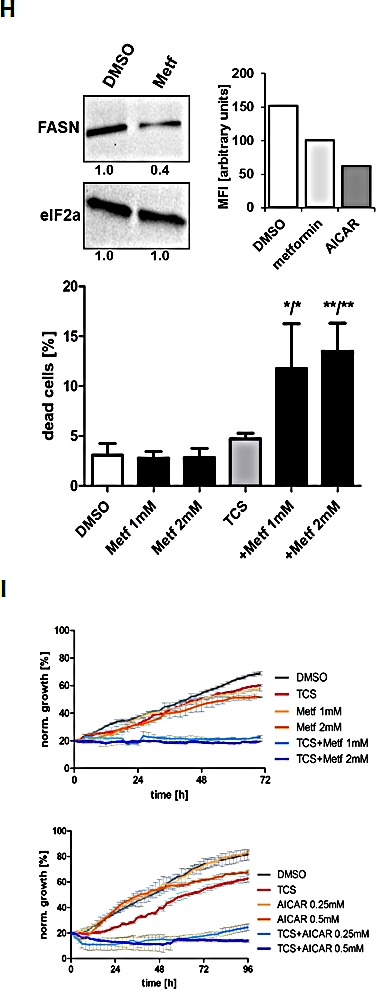
TCS causes G0/G1 cell cycle arrest and induces apoptosis in PCa cells (A) LNCaP cells were treated with control (DMSO), 10 μM TCS, 40 μM C75 or 40 μM orlistat for 48 h, and cell cycle distribution was analyzed by flow cytometry and quantitated with ModFit LT. (B) PC-3 cells were treated with the indicated concentrations of TCS for 48 h and analyzed as in A. The results are representative of three independent experiments. (C) LNCaP cells were treated for 48 h with the indicated concentrations of TCS analyzed by flow cytometry. The percentage of dead cells with hypodiploid subG1 DNA content was quantitated with ModFit LT (n=3, mean±SD, *p<0.05, **p<0.01). (D) LNCaP cells were treated with 20 μM TCS for the indicated times and analyzed as in C. (E) LNCaP were treated with the indicated concentrations of TCS for 48 h, and apoptosis was analyzed by co-staining with Annexin V-FITC and PI. Early apoptotic cells (Annexin V-positive and PI-negative cells) were quantitated by flow cytometry (n=3, mean±SD, *p<0.05, **p<0.01). (F) LNCaP cells were treated as in E, and PARP cleavage was detected by immunoblotting. (G) FASN expression and lipogenesis were stimulated by treating androgen-deprived LNCaP cells (black bars) with 10 nM DHT (grey bars) or 5% FBS (white bars) prior incubation with the indicated concentrations of TCS for 48 h. The number of apoptotic cells was quantitated as in C. (H) Activation of AMPK decreases FASN expression and cellular lipid levels. Western blot analysis of FASN expression in LNCaP cells after treatment with 2 mM metformin for 48 h. As a loading control, protein levels of eIF2 were determined. Cellular lipid levels of LNCaP cells treated with 2 mM metformin or 0.5 mM AICAR for 48 h were measured by Nile Red staining and flow cytometry. LNCaP cells were incubated with the indicated concentrations of TCS for 48 h in the absence (white bars) or presence of metformin (black bars). Cell death was measured as in C (n=3, mean±SD, *p<0.05, **p<0.01). (I) LNCaP cells were treated with 5 μM TCS, 1-2 mM metformin or a combination of both compounds, and cell confluence was measured every 2 h for 72 h (top panel). The co-treatment of LNCaP cells was repeated with 7.5 μM TCS and 0.25-0.5 mM AICAR for 96 h (bottom panel).

We next performed DNA content analysis by FACS to measure the effect of different concentrations of TCS, C75 and orlistat on the cell cycle distribution of asynchronously growing LNCaP cells after 48 h of treatment. Similar to previous studies in RKO colon cancer cells [36] and A-375 melanoma cells [37], C75 increased the number of LNCaP cells in S phase and G2/M of the cell cycle (Fig. 3A). We observed similar results in PC-3 and LAPC4 cells (data not shown). Importantly, Ho *et al.* showed that the G2/M arrest of A-375 melanoma cells generated by C75 preceded induction of apoptosis and DNA fragmentation [37], suggesting that C75 is cytotoxic in proliferating cells. Consistent with this, C75 was more potent in fast growing PCa cell lines (PC-3 and 22RV1) when compared to slow growing LNCaP cells (Table 2). Unlike C75, TCS (10 μM) and orlistat (40 μM) led to an increase in the number of cells in G0/G1 and, concomitantly, a reduction of S phase and G2/M cells (Fig. 3A), suggesting a cell cycle arrest in G0/G1, which was previously reported for orlistat in the breast cancer cell line MDA-MB-435 [38]. Consistent with the above findings in LNCaP cells, TCS also caused a G0/G1 cell cycle arrest in PC3 (Fig. 3B) and 22RV1 cells (data not shown).

Compared to the control, treatment with 20 μM TCS caused a 12-fold increase in the number of LNCaP cells in the hypodiploid subG1 peak, which is indicative of DNA fragmentation and cell death (Fig. 3A). For comparison, 30 μM C75 and 40 μM orlistat increased the number of cells in subG1 by six-fold and 11-fold, respectively (Fig. 3A). Further subG1 analysis of titration (Fig. 3C) and time course experiments with LNCaP cells (Fig. 3D) showed that TCS induced cell death in a concentration- and time-dependent manner. Similar results were observed in PC-3 cells (data not shown). Importantly, FACS analysis of the apoptosis marker phosphatidylserine with Annexin V confirmed that TCS induced cell death through apoptosis in a concentration-dependent manner in LNCaP (Fig. 3E) and PC-3 cells (data not shown). Furthermore, immunoblotting with poly(ADP-ribose) polymerase (PARP) antibody showed that TCS caused PARP cleavage in LNCaP cells (Fig. 3F). Together, these results demonstrate that TCS causes a G0/G1 cell cycle arrest and induces apoptosis in PCa cells.

Consistent with the above findings that TCS caused cytotoxicity predominantly through end product starvation (Fig. 1F), androgen deprivation (Fig. 3G) or inhibition of androgen signalling with the androgen receptor antagonist bicalutamide (data not shown), which reduces expression of the androgen receptor-regulated FASN gene and lipogenesis [39, 40], increased the sensitivity of LNCaP cells to TCS, as shown by a significant increase in dead cells in the hypodiploid subG1 population when compared to cells grown in the presence of the androgen dihydrotestosterone (DHT) or 5% fetal calf serum (Fig. 3G). Similar results were observed in androgen-sensitive 22RV1 cells (results not shown). Similarly, co-treatment with the AMP-activated protein kinase (AMPK) activator metformin, which leads to a robust reduction of FASN expression and cellular lipid levels (Fig. 3H) via suppression of the lipogenic transcription factor SREBP1 [28], enhanced the cytotoxic effect of TCS in LNCaP (Fig. 3H) and 22RV1 cells (data not shown), as indicated by a substantial increase in dead cells (hypodiploid subG1 population) when compared to TCS or metformin treatment alone. Consistent with this, real-time live cell imaging demonstrated that co-treatment of LNCaP cells with TCS and the AMPK agonists metformin or AICAR substantially enhanced the cytotoxic effect of TCS when compared to the individual treatments (Fig. 3I). Taken together, these results indicate that the levels of FASN expression and lipogenesis are strongly correlated with sensitivity of PCa cells to TCS.

### Triclosan reduces the lipid content of LNCaP cells

**Figure 4:**
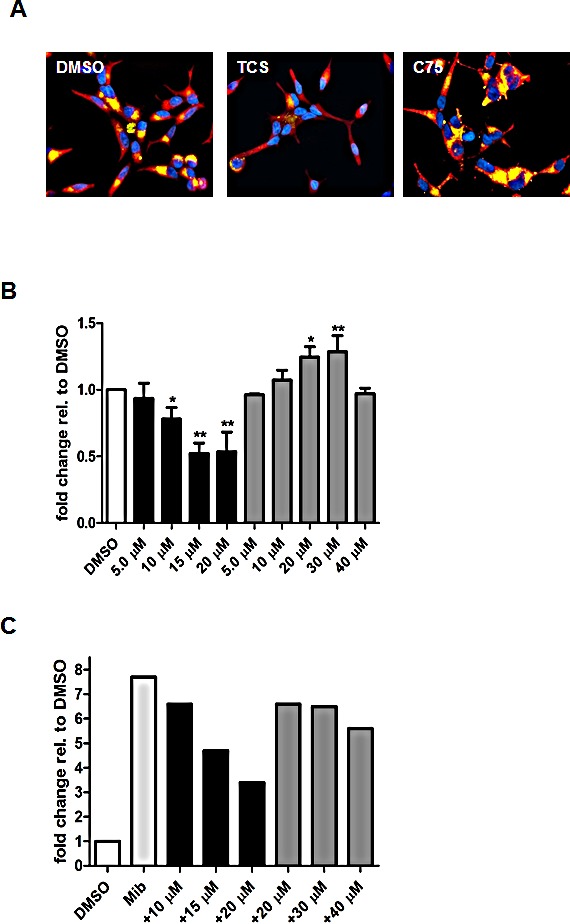
Triclosan reduces the lipid levels of LNCaP cells (A) LNCaP cells treated with 10 μM TCS or 30 μM C75 for 48 h. Neutral (yellow) and polar lipids (red) were labelled with Nile Red and visualized by fluorescence microscopy. DNA was counterstained with DAPI (blue). (B) LNCaP cells were grown in 5% FBS before treatment with the indicated concentrations of TCS (black bars) or C75 (gray bars) for 48 h. After fixation, cellular lipids were stained with Nile Red, and fluorescence of neutral lipids was measured in a plate reader (n=3 ±SD, *p<0.05, **p<0.01). (C) FASN expression and lipogenesis were stimulated by treating androgen-deprived LNCaP cells (white bar) with 1 nM mibolerone (light gray bar) for 72 h prior incubation with the indicated concentrations of TCS (black bars) or C75 (dark gray bars) for 48 h. Samples were processed and measured as in B.

To evaluate the effect of FASN inhibition by TCS or C75 on the cellular lipid content, we stained LNCaP cells with the lipophilic fluorescent dye, Nile Red. The fluorescence intensity of Nile Red-stained cells is linearly correlated with the lipid content [41]. Nile Red displays different emission maxima, depending on the hydrophobicity of the bound lipids [42]. For example, when excited at 485 nm, Nile Red-stained neutral lipids like triacylglycerols and cholesterol esters, which are the main contents of lipid droplets, fluoresce with an emission maximum of ~520 nm, while polar lipids like phosphatidylcholine, phosphatidylethanolamine and phosphatidylserine, which are the main constituents of lipid bilayers, have an emission maximum of ~620 nm. As shown in Figure 4A, fluorescence microscopy revealed that treatment of LNCaP cells with 10 μM TCS visibly reduced the staining of neutral and polar lipids when compared to control or cells treated with 30 μM C75 (Fig. 4A). Quantitative analysis of Nile Red-stained lipids in LNCaP cells indicated that increasing concentrations of TCS lowered the neutral and polar lipid content by up to two-fold (Fig. 4B). This was in stark contrast to the biphasic effect seen with C75, where increasing concentrations from 5-30 μM caused a steady rise of the lipid content, while C75 at 40 M reversed this stimulatory effect. We repeated the measurement of cellular lipids with LNCaP cells in which FASN expression and neutral lipid levels were markedly upregulated through stimulation of lipogenesis with the synthetic androgen mibolerone [39, 40] prior to the addition of TCS (Fig. 4C). When compared to control, androgen treatment increased the neutral lipid content by almost 8-fold. Incubation with 10-20 μM TCS caused a decline in neutral lipids of up to 2.5-fold, while only a modest reduction in the lipid content was detected when treated with 40 μM C75 (Fig 4C). Taken together, these results demonstrated that the FASN inhibitor TCS reduced the lipid content of LNCaP cells in a concentration-dependent manner.

### The hydroxyl group of triclosan is critical for its cytotoxicity

**Figure 5:**
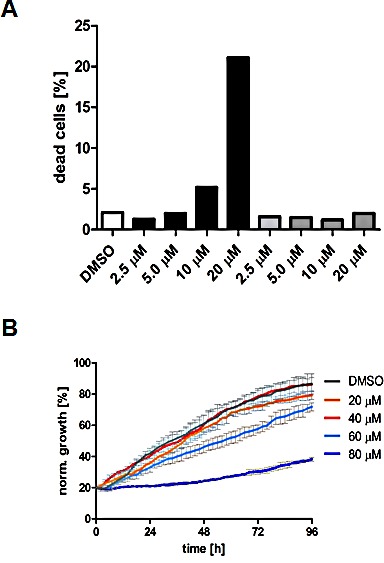

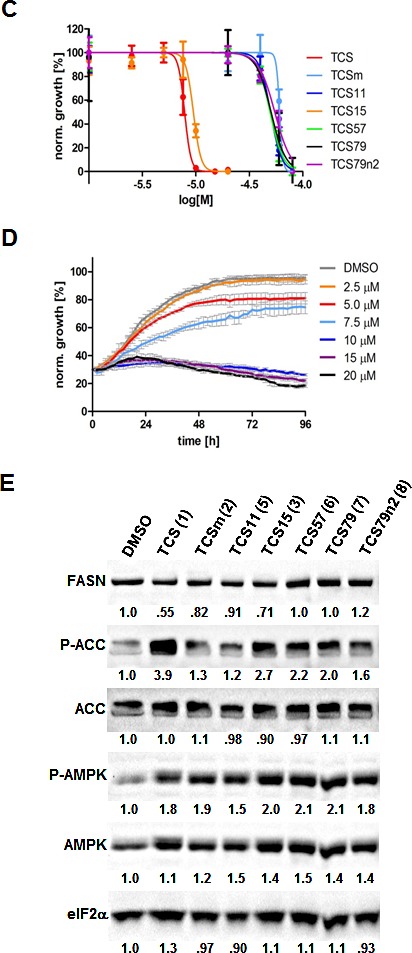
The hydroxyl group of triclosan is critical for its cytotoxic activity (A) LNCaP cells were treated for 48 h with control (DMSO) or increasing concentrations of TCS (black bars) or its derivative TCSm (gray bars). Samples were analyzed by flow cytometry, and the percentage of apoptotic cells with hypodiploid subG1 DNA content was quantitated with ModFit LT. (B) LNCaP cells were treated with control (DMSO) or the indicated concentrations of TCSm, and confluence was measured every 2 h for 96 h (n=3 ±SD). (C) LNCaP cells were treated with increasing concentrations of the indicated TCS analogs and monitored by live cell imaging as described in B. Non-linear regression analysis of the dose response for 48 h is shown. (D) LNCaP cells were treated with control (DMSO) or the indicated concentrations of TCS15, and growth was measured as described in B (n=3 ±SD). (E) Western blot analysis of key lipogenic and energy sensing enzymes. LNCaP cells were treated for 24 h with control (DMSO) or 10 μM of the indicated TCS analogs, and protein lysates were probed with antibodies directed against the indicated proteins. For quantification, total protein levels were normalized relative to loading control (eIF2α). The level of protein phosphorylation was calculated relative to the normalized total protein amount.

We next tested the commercially available triclosan derivative, methyl triclosan (TCSm, 2), in which the hydroxyl group of the phenol is replaced by a methyl ether group (Table 3). As shown in Figure 5A, FACS analysis of the hypodiploid subG1 cell population revealed that equimolar concentrations of TCSm (2) failed to induce cell death in LNCaP cells after 48 h of treatment when compared to TCS. We observed similar results in the 22RV1 cell line (results not shown). Furthermore, live cell analysis of cell confluence (IncuCyte) demonstrated that TCSm (2) reduced cell growth of LNCaP cells at concentrations higher than 20 μM, but no cytotoxicity was detected at concentrations of up to 80 μM (Fig 5B). Similar results were obtained by Alamar Blue assay (Table 3). Kinetic analysis of the IncuCyte and Alamar Blue data for TCSm treatment of LNCaP cells for 48 h with revealed an IC50 of 52.6 μM and 60.8 μM (Table 3), respectively, representing an 8 to 9-fold reduction in potency when compared to TCS (6.9 μM and 6.6 μM). We also noticed that TCSm did not induce the above described changes to cell morphology, e.g. flat and enlarged cells with increased granularity (results not shown), and 20 μM TCSm did not reduce the neutral and polar lipid content of LNCaP cells (Table 3). These results suggested that the hydroxyl group of triclosan may be critical for its potency as a FASN inhibitor, and prompted us to generate five derivatives for preliminary structure-activity relationship studies (Table 3). The IC50 of the TCS analogs were determined in LNCaP cells by live cell imaging (IncuCyte) and Alamar Blue assay, and cellular lipid levels were measured by Nile Red staining (Table 3). All analogs but TCS15 (3) showed substantial losses in cytotoxic potency (Fig. 5C) and the ability to reduce cellular lipid levels (Table 3), respectively. Interestingly, LNCaP cells displayed a biphasic dose response profile when treated with 10-20 μM TCS15 (Fig 5D), where, unlike the parent compound (Fig. 1A), cytotoxicity was observed with a delay of 24 h post treatment. We suggest that the inhibitory effect of TCS15 can be explained by hydrolysis of the phenolic acetate to release the parent compound in the biological system. Changes in alcohol pKa and spacing substantially reduced activity, as shown for analog TCS57 (6), which bears a chain-extended benzylic alcohol in place of the parent phenol. Similarly, compounds TCS79 (7) and TCS79n2 (8), which bear chain-extended ester and carboxylic acid functionality, respectively, did not maintain the inhibitory activity of TCS. Analogs TCSm (2) and TCS11 (5), which lack alcohol functionality showed very limited inhibitory activity. Together, these results strongly suggest that the activity of TCS is dependent on alcohol functionality of the parent phenol.

**Table:** Structure-function relationship analysis of TCS analogs: Cytotoxic potency and relative lipid levels

analog	structure	IC50 [μM] IncuCyte	IC50 [μM] Alamar Blue	Neutral Lipids*	Polar Lipids*
TCS (1)	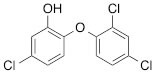	6.9	6.6	0.40	0.19
TCSm (2)	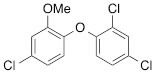	52.5	60.8	0.97	1.09
TCS11 (5)	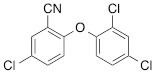	44.8	50.0	0.70	1.06
TCS15 (3)	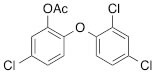	9.3	9.5	0.56	0.60
TCS57 (6)	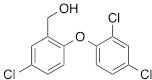	34.5	49.3	0.90	0.81
TCS79 (7)	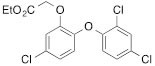	44.8	50.1	1.29	1.13
TCS79n2 (8)	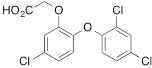	56.3	54.7	1.10	1.03

* Lipid content relative to DMSO at 20 μM for 48 h

As shown above, inhibition of FASN by TCS increased the levels of phosphorylated AMPK and ACC and decreased FASN protein levels (Figs. 1D and 1E). To determine if these molecular effects were correlated with the cytotoxic effect of the TCS analogs, we treated LNCaP cells for 24 h with 10 μM of the TCS analogs and analyzed FASN, ACC and AMPK expression by Western blot analysis (Fig. 5E). Notably, among the five analogs, only TCS (Fig. 1A) and TCS15 (3) (Figs. 5C and 5D) inhibited growth of LNCaP cells at this concentration. As shown in Figure 5E, all TCS analogs caused a similar increase in AMPK phosphorylation (1.5 to 2.1-fold), whereas phosphorylation of ACC was increased the most by TCS (3.9-fold) followed by TCS15 (2.7-fold). Similarly, FASN expression was decreased the most by TCS (-1.8-fold) and TCS15 (-1.4-fold), while the less cytotoxic analogs caused only a modest change in FASN levels (-1.2 to 1.2-fold). Taken together, these results suggest that TCS-induced cytotoxicity is linked to a reduction in FASN protein levels but does not correlate with the level of AMPK phosphorylation. In summary, these results demonstrated that the hydroxyl group is critical for the activity of TCS in inhibiting cancer cell growth and reducing cellular lipid levels and FASN expression.

## DISCUSSION

TCS has U.S. Food and Drug Administration (FDA) approval as a bactericide in personal hygiene products (toothpaste, mouth rinse, handwash, soaps, deodorant) and has been used since 1968. TCS has an established safety profile, with minimal toxicity in rats, dogs, baboons and humans, no significant weight loss is associated with TCS treatment, and TCS is not a genotoxic or mutagenic compound. TCS has excellent oral bioavailability and stability in plasma (reviewed in [43]). Promising results have shown previously that FASN inhibition by TCS showed chemo-preventative activity in a rat mammary carcinogenesis model [19]. In this study we evaluated TCS, which inhibits the enoyl reductase domain of FASN [8], as a repurposed drug against PCa cells and compared its activity to C75 and orlistat, two well-known FASN inhibitors whose clinical development are impeded by various pharmacological limitations (reviewed in [44]). Orlistat, a U.S. FDA-approved drug designed for obesity, inhibits pancreatic lipase, thereby preventing the absorption of free FAs from the diet in the intestine. Orlistat is also an irreversible inhibitor of the thioesterase activity of FASN and decreases cell viability of PCa cells by inducing apoptosis [29]. Its potential application for cancer chemotherapy is limited by its poor solubility and very low level of oral bioavailability (reviewed in [44]). C75 is a synthetic analog of cerulenin and lacks the unstable, highly reactive epoxy group [9]. FASN inhibition by C75 induces apoptosis [45] and showed significant antitumor activity with little adverse effects on normal proliferating tissues, such as bone marrow, intestine, skin, or lymphoid tissue [9]. However, studies in mice revealed that C75 causes strong appetite suppression and profound weight loss through direct activation of carnitine O-palmitoyltransferase (CPT-1), thereby increasing FA β-oxidation [10]. With regards to mechanism of action, C75 is a unique FASN inhibitor since it targets the first reaction step which is catalyzed by the β-ketoacyl synthase partial activity, as well as inactivates the TCS-targeted enoyl reductase and orlistat-targeted thioesterase partial activities with similar rates [5].

Like C75 and orlistat [29, 45], FASN inhibition by TCS ultimately caused cell death of LNCaP cells via apoptosis in a concentration- and time-dependent manner (Figs. 3A-3F). TCS was cytotoxic in five different PCa cell lines with similar IC50s in the low micromolar range (4.5-7.8 μM). Early structure-function relationship analysis with a panel of seven TCS analogs suggested that alcohol functionality of the parent phenol is critical for cytotoxic potency (Fig. 5 and Table 3). In comparison, C75 displayed cytotoxic potency that varied by more than four-fold between PCa cell lines (8.3-35.4 μM, Table 2) and appeared to be correlated with the proliferation rate / number of cells in S phase (data not shown). Indeed, previous work has shown that C75 induces apoptosis of MCF7 and A-375 during S phase [37], suggesting that C75 is more potent in rapidly proliferating cancer cells. Similar to previous reports [18], we found that TCS and C75 were non-cytotoxic at up to 60 μM in non-malignant NIH3T3 fibroblasts. Yet, our analysis showed considerable cytotoxicity of TCS and C75 in the non-malignant prostate cell lines BPH-1, RWPE-1 and WPMY-1, with IC50s ranging between 0.74 M and 28.2 μM (Table 2). A recent study described a similar observation, showing that RWPE-1 cells were more sensitive to FASN inhibition by siRNA or C75 than the malignant cell lines LNCaP, DU145 or PC-3 [46]. Furthermore, TCS has been shown to induce degeneration and atrophy of prostatic tissue in rats after treatment for 60 days with a daily dose of 20 mg/kg [20]. Taken together, our observations suggest that cells of prostatic origin are particularly sensitive to FASN inhibition. We found no correlation between FASN inhibitor sensitivity and the status of the androgen receptor, PTEN, or p53 in the cell lines tested (results not shown).

Real-time monitoring of FASN inhibitor cytotoxicity by IncuCyte technology based on cell confluence provided valuable kinetic information about potency, growth inhibition and cell death (Fig. 1A). These observations were validated by Alamar Blue assay which measures cell viability based on metabolic activity (Table 1). Interestingly, inhibition of the rate-limiting enzyme of *de novo* FA synthesis, ACC, with TOFA or FASN with TCS reached maximum potency in LNCaP cells as early as 6 h after treatment commenced and remained relatively unchanged over the entire incubation period (Fig. 1C). This is in agreement with a significant rise in cell death of LNCaP cells when treated for >6 h with TCS (Fig. 3D). In contrast, the potency of C75 (Fig. 1C) and orlistat (data not shown) to inhibit growth of LNCaP cells changed by two-fold over the treatment period and required up to 72 h to reach maximum potency. Importantly, this analysis demonstrated that there were substantial differences between the four inhibitors of *de novo* FA synthesis (TOFA, TCS, C75 and orlistat) with regards to growth profile and timing of growth inhibition at IC50 (Fig. 1A) and time-dependent maximum potency (Fig 1C), suggesting that they induced cytotoxicity through different mechanisms, e.g., end product starvation or accumulation of toxic levels of malonyl CoA substrate or intermediates of palmitate synthesis. Indeed, rescue experiments with exogenous palmitate showed that TCS- and orlistat-induced cytotoxicity was largely caused by end product starvation (Fig. 1G). This mechanism of cytotoxicity was previously reported for orlistat in PC-3 cells [29]. Consistent with earlier work [30], C75-induced growth inhibition of LNCaP cells could not be rescued by palmitate (Fig. 1G), and it was suggested previously that accumulation of toxic levels of malonyl-CoA caused cytotoxicity of C75 [31]. This is a particularly interesting observation, given that (i) C75 inactivates three partial activities of FASN (β-ketoacyl synthase, enoyl reductase and thioesterase) with similar rates [5], (ii) inhibition of the enoyl reductase activity by TCS and thioesterase activity by orlistat cause cytotoxicity predominantly through end product starvation (Fig. 1G) and (iii) TCS-mediated inhibition of the enoyl reductase activity was suggested to increase the concentration of the enoyl thiolester intermediate, which strongly resembles the two β-ketoacyl reductase inhibitors, CM-55 and C75 [8]. Hence, it will be interesting to address biochemically in future work how inactivation of various partial catalytic activities of FASN contribute to overall cytotoxicity through accumulation of potentially toxic metabolites.

In addition to the cause of cytotoxicity by end product starvation, TCS-mediated inhibition of FASN generated phenotypes in LNCaP cells with regards to cell morphology (Fig. 1B), cell cycle (Fig. 3) and expression of FASN protein (Fig. 1D) that resembled the effects seen with orlistat, which were distinctively different to C75. Of note was our discovery that TCS caused a significant reduction in FASN gene (Fig. 2) and protein expression (Figs. 1D and 1E) in LNCaP cells, while C75 led to an increase. FASN gene expression was also increased by orlistat and TOFA. Yet, gene expression of the lipogenic transcription factor SREBP1 remained unchanged by the FASN inhibitors TCS, C75 and orlistat, while it was increased two-fold by TOFA. In addition, we discovered that the lipogenic inhibitors caused distinct changes to the expression of genes involved in FA uptake and transport (SLC27A1 and ACBP), FA conversion and remodeling (HSL/LIPE, ACSL5 and FADS-2), FA β-oxidation (CPT-1), and arachidonic acid metabolism (PLA2G6 and PTGS1) (Fig. 2), suggesting the activation of different feedback mechanisms. This was further exemplified by the significant upregulation of key genes of cholesterol synthesis (SREBP2, HMGCS and HMGCR) by orlistat and TOFA but not TCS and C75.

We also observed that inhibition of *de novo* FA synthesis with four different inhibitors targeting ACC (TOFA) and FASN (TCS, C75, orlistat) caused a substantial increase in AMPK phosphorylation. Yet, siRNA-mediated knockdown of FASN protein by 94% after 96 h did not change AMPK phosphorylation (data not shown). Furthermore, the levels of AMPK phosphorylation did not correlate with cytotoxic potency, FASN inhibition and reduction of cellular lipids, as shown by the inactive TCS analogs (Table 3). These observations suggest that AMPK phosphorylation was not directly induced by a reduction in *de novo* FA synthesis, and that other canonical or non-canonical pathways might be responsible for AMPK activation, e.g. xenobiotic or oxidative stress (reviewed in [25, 47]). Furthermore, while metformin- and AICAR-induced upregulation in AMPK phosphorylation caused an expected and proportional increase in ACC phosphorylation, we noticed that this was not the case with the lipogenic inhibitors (Fig. 1D).

Previous work has shown that 7.9–39.4 μM C75 substantially reduced incorporation of [U-^14^C]acetate via inhibition of *de novo* synthesis into acylglycerides (80%) and FAs (50%) in HL60 cells without affecting phospholipid synthesis [9]. Interestingly, C75 caused a biphasic effect on total neutral and polar lipid content (Fig. 4), which is the sum of lipids derived from *de novo* synthesis and recycling pathways. 5-30 μM C75 increased the neutral lipid content of LNCaP cells, while 40 μM C75 suppressed this stimulation to base line levels (Fig. 4B). Previous work in CHO-K1 cells has shown that C75-induced cellular stress caused lipid droplet biogenesis and metabolic synthesis of triacylglycerol through recycling of structural phospholipids [48]. In contrast, TCS reduced cellular lipid levels in a concentration-dependent manner (Figs. 4B and 4C), suggesting that TCS affected the FA recycling pathway differently. Furthermore, preliminary structure-function relationship studies with seven TCS analogs demonstrated that the reduction of cellular lipid levels was correlated with cytotoxic potency and dependent on alcohol functionality of the parent phenol, respectively.

Androgen ablation therapy (ADT) has been the standard treatment for metastatic PCa for decades, and treatment of type II diabetes with the AMPK activating drug metformin has been shown to improve patient outcomes in PCa (reviewed in [49]). Moreover, a recent study of the novel AMPK activator MT 63-78 provided a rationale for blocking lipogenesis through the combined targeting of AMPK and the androgen receptor in advanced PCa [50]. Consistent with these reports, we found that ADT and treatment with the AMPK activators metformin and AICAR, which cause a reduction of FASN gene and protein expression and lipogenesis, sensitized LNCaP cells to FASN inhibition by TCS (Figs. 2G-2I), suggesting that combination therapy of FASN inhibitors with ADT and/or AMPK activators (metformin, AICAR, MT 63-78) might improve the clinical outcome of PCa patients.

In this comparative study we discovered that TCS is a superior alternative to C75 and orlistat in inducing cell death of PCa cells via inhibition of FASN. Strikingly, inhibition of different partial catalytic activities of FASN generated distinct phenotypes with regards to cell morphology, cell cycle, total lipid content, and the expression of FASN and key genes of FA and cholesterol metabolism, highlighting the need for further studies of the mechanism of action of the cytotoxicity-inducing molecule(s), i.e. malonyl-CoA, intermediates of FA synthesis, depletion of palmitate. The known safety profile of TCS and its chemo-preventative antitumorigenic activity warrant further characterization of TCS in a pre-clinical setting to address its efficacy in inhibiting PCa xenograft tumor growth. Thus, TCS-mediated inhibition of the metabolic oncogene FASN has promising potential for therapeutic development for advanced PCa.

## MATERIAL AND METHODS

### Reagents

Triclosan [5-chloro-2-(2,4-dichlorophenoxy)phenol] (1) (Merck Calbiochem), methyl triclosan [5-chloro-2-(24-dichlorophenoxy)anisole] (2) (Sigma), C75 [(2R*,3S*)-Tetrahydro-4-methylene-2-octyl-5-oxo-3-furancarboxylic acid] (Sigma, Tocris Bioscience), orlistat [N-formyl-L-leucine (1S)-1-[[2S,3S)-3-hexyl-4-oxo-2-oxetanyl]methyl]dodecyl ester] (Sigma), AICAR [5-amino-1-β-D-ribofuranosyl-1H-imidazole-4-carboxamide] (Sigma), TOFA [**5-(Tetradecyloxy)-2-furoic acid]** (Sigma) were dissolved in DMSO, metformin [1,1-dimethylbiguanide hydrochloride] (Sigma) was dissolved in phosphate-buffered saline (PBS), Nile Red (Sigma) was dissolved in acetone and diluted in PBS, the androgens dihydrotestosterone (DHT) (Sigma), mibolerone (a kind gift from Dr. L. Butler) and R1881 (Dupont) as well as palmitate (Sigma) were dissolved in 95% ethanol (EtOH) and diluted in 20% ethanol.

### Cell culture

LNCaP, C4-2B, PC-3, 22RV1, and RWPE-1 cells were obtained from the American Type Cell Culture Collection. LAPC4, BPH-1, WPMY-1, and 3T3 cells were kind gifts from P. J. Russell, J. Clements and J. Whitehead, respectively. LNCaP, C4-2B, PC-3, 22RV1, BPH-1, and WPMY-1 cells were maintained in phenol-red free RPMI-1640 medium (Invitrogen) supplemented with 5% fetal calf serum (FBS) (Invitrogen) at 37°C in an atmosphere containing 5% CO_2_. LAPC4 cells were grown in Iscove's Modified Dulbecco's Medium (IMDM) (Invitrogen) supplemented with 5% FBS and 10 nM DHT. RWPE-1 cells were maintained in Keratinocyte-SFM medium (Invitrogen), and 3T3 cells were grown in Dulbecco's Modified Eagle Medium (DMEM, Invitrogen) supplemented with 10% newborn calf serum (NCS, Invitrogen). For androgen-mediated upregulation of FASN expression and lipogenesis [39, 40], cells were grown after seeding in phenol-red free RPMI-1640 with 5% FBS for an additional 48 h in phenol-red free RPMI-1640 supplemented with 5% charcoal-stripped serum (CSS) (Invitrogen). After this, medium was supplemented with androgens (1 nM mibolerone or 10 nM DHT), and cells were grown for another 72 h before treatment with TCS or C75 for 48 h.

### Live imaging and Alamar Blue assay

Cells were seeded in 96-well plates at 4.0 × 10^3^ (LNCaP, C4-2B, 22RV1, LAPC4) or 3.0 × 10^3^ cells per well (PC-3, RWPE-1, WPMY-1, BPH-1 and NIHT3T) and grown to 20% confluence before addition of the indicated compounds. For rescue experiments, palmitate (Sigma) was conjugated with FA-free BSA (Roche Applied Science) at a 6:1 ratio for 1 h at 37°C and diluted into growth medium. Growth as a function of increasing confluence was measured in real-time by phase contrast microscopy with the IncuCyte FLR system (Essen BioScience). Images were taken with a 10x objective at 2 h intervals from 3 separate wells per treatment, and mean ±SD of confluence percentages was computed. For Alamar Blue endpoint assays, cells were seeded into 96-well tissue culture plates and treated with the indicated compounds as described above. Metabolic activity was measured with Alamar Blue after 48 h of treatment according to the manufacturer's instruction (Invitrogen, USA). Kinetic analysis and calculation of IC50 were performed with GraphPad Prism (GraphPad Software).

### Fluorescence microscopy

LNCaP cells were seeded in 24-well plates on cover slips at 1.0 × 10^4^ in phenol-red free RPMI-1640 medium supplemented with 5% FBS before treatment with TCS for 48 h. Cells were washed with 1 ml PBS, fixed for 20 min in 4% paraformaldehyde on ice and washed again with 1 ml of PBS. Finally, cellular lipids were stained with 0.1 μg/ml Nile Red (Sigma), and nuclear DNA was counterstained with 1 μg/ml 4′,6-diamidino-2-phenylindole (DAPI) (Invitrogen). Cells were imaged on a Nikon Eclipse Ti fluorescent microscope equipped with NIS Elements imaging software (Nikon). Fluorescence of Nile Red was excited at 488 nm, and emission was acquired at 530 nm with a FITC filter and at 610 nm with a Texas Red filter.

### Lipid content

Cells were seeded at 2.0 × 10^5^ cells per well in 6-well plates in phenol-red free RPMI-1640 medium supplemented with 5% FBS for 48 h. After treatment for 48 h with TCS or C75, cells were harvested with trypsin, washed with 1 ml of PBS and fixed in 4% paraformaldehyde for 20 min on ice. Cells were washed again with 1 ml of PBS and finally resuspended in 0.2 ml of PBS. For androgen treatment, cells were seeded at 8.0 × 10^4^ cells per well in 6-well plates and processed as described above. A 96-well plate was set up with 40 μl of cell suspension in a total volume of 100 μl with PBS, and lipids were stained with Nile Red at a final concentration of 0.5 μg/ml. For normalisation, 40 μl of cell suspension in a total volume of 100 μl with TE (10 mM Tris-HCl pH 7.5, 1 mM EDTA) was mixed with 0.5 μl of Quant-iT PicoGreen DNA stain (Invitrogen) according to the manufacturer's instructions. Fluorescence of Nile Red-stained neutral lipids (485 nm/520 nm) and polar lipids (485 nm/620 nm) and Quant-iT PicoGreen-stained DNA (485 nm/520 nm) was measured in a FLUOstar Omega plate reader (BMG Labtech). Fluorescence intensities were normalized according to the DNA content and calculated as fold-changes relative to control. For analysis by flow cytometry, cells were seeded and harvested as described above and directly stained with Nile Red (0.5 μg/ml). The median fluorescence intensity of 20,000 cell events was measured at 520 nm with a FITC filter on a FACSCanto (BD Biosciences).

### Western blot

Cells were plated at 1.5 × 10^5^ cells per well in 6-well plates in phenol-red free RPMI-1640 medium supplemented with 5% FBS for 48 h and treated for the indicated times. Cells were lyzed in 120 μl RIPA buffer supplemented with protease and phosphatase inhibitors as described previously [51]. Cell lysates (30ug/ lane) were separated by SDS-polyacrylamide gel electrophoresis, and proteins were transferred to PVDF membrane (Millipore). Primary antibodies were from Cell Signaling Technologies and used as recommended by the manufacturer; FASN (#3189), ACC (#3676), P(Ser79)-ACC (#3661), PARP (#9542), AMPKα (#2793), P(Thr172)-AMPKα (#2531), eIF2α (#2103). Beta-Actin antibody (sc-47778, Santa Cruz Biotechnology) or eIF2α (#2103, Cell Signaling Technologies) were used to normalize protein loading. PVDF membranes were probed with the appropriate horseradish peroxidase-conjugated secondary antibody (GE Healthcare) and visualized with a chemiluminescence reaction system (Immobilon Western Chemiluminescent HRP Substrate, Merck Millipore) and documented on a ChemiDoc XRS system (Bio-Rad). Proteins were quantitated using Image Lab™ software (Bio-Rad), normalized to the respective loading control, and expressed relative to the control treatment. Phosphorylation levels were calculated relative to the normalized total amount of the respective protein.

### Flow cytometry

Cells were seeded at 2.0 × 10^5^ cells per well in 6-well plates in phenol-red free RPMI-1640 medium supplemented with 5% FBS for 48 h. After treatment for 48 h with TCS or C75, cells were harvested with trypsin, washed with 1 ml of PBS and fixed in 1 ml of 70% ethanol for 60 min at −20°C. Cells were washed in 1 ml of PBS, resuspended in 0.5 ml of PBS supplemented with 10 μg/ml propidium iodide (PI) (Sigma) and 30 μg/ml DNAse-free RNAse A (Sigma) and incubated over night at 4°C. Samples were analyzed on a FACSCanto (BD Biosciences), where 20,000 events were counted after exclusion of cell doublets and polyploid cell populations. DNA histograms were analyzed with ModFit LT (Verity Software House). For the detection of apoptosis, samples were prepared as above except that cells were not fixed in 70% ethanol. Instead, 1 × 10^5^ cells were resuspended in 1 x Annexin V binding buffer (10 mM Hepes-NaOH pH 7.4, 150 mM NaCl, 2.5 mM CaCl_2_) containing 2 μl of 0.15 mg/ml FITC-conjugated Annexin V (BioVision) and PI at 5 μg/ml and incubated for 5 min in the dark. After electronic compensation, FITC and PI fluorescent intensities were measured for 20,000 cell events on a FACSCanto (BD Biosciences) and analyzed with FACSDiva software (BD Biosciences).

### Quantitative real-time PCR

LNCaP cells were seeded at a density of 1 × 10^5^ cells/well in a 6-well plate. After 48 h, cells were treated with the indicated inhibitors. After an additional 48 h, total RNA was obtained using the RNeasy mini kit (Qiagen) according to the manufacturer's instructions. cDNA was prepared from 2 μg total RNA with Superscript III (Invitrogen). qRT-PCR was performed with SYBR Green PCR Master Mix (Invitrogen) on a 7900HT Fast Real-Time PCR System (Applied Biosystems). The mRNA expression levels were calculated according to the ΔΔCt method and normalized relative to the expression levels of the house keeping gene (RPL32) of the respective treatment and expressed as fold change relative to the control (DMSO). Statistical significance was analyzed with GraphPad Prism (GraphPad Software) by one-way ANOVA with Bonferroni post-tests. The sequences of the primers used are listed in the Supporting Information.

### Synthesis of TCS derivatives

The chemical synthesis and validation of the TCS derivatives is described in the Supplemental Material.

## SUPPLEMENTARY MATERIAL



## Abbreviations

TCS: triclosan
FASN: fatty acid synthase
FA: fatty acid
AMPK: 5′-AMP-activated protein kinase
Metf: metformin
Mib: mibolerone
PCa: prostate cancer
