# Supplementary material for: The fatty acid synthase inhibitor triclosan: repurposing an anti-microbial agent for targeting prostate cancer
Source: Oncotarget. 2014 Sep 3;5(19):9362–81. doi: 10.18632/oncotarget.2433 (PMC4253440; doi:10.18632/oncotarget.2433)

The fatty acid synthase inhibitor triclosan: repurposing an anti-microbial agent for targeting prostate cancer

Supplementary Material

Appendix A. Supplementary data

The following are supplementary data related to this article.

Primer sequences used for qRT-PCR

Gene	Primer	Sequence
SREBP1	forward<br>reverse	5’-CGCTCCTCCATCAATGACAA-3’<br>5’-TCGAGAAAGCGAATGTAGTCGAT-3’
ACC	forward<br>reverse	5’-CTGTAGAAACCCGGACAGTAGAAC-3’<br>5’-GGTCAGCATACATCTCCATGTG-3’
FASN	forward<br>reverse	5’-CGCTCGGCATGGCTATCT-3’<br>5’-CTCGTTGAAGAACGCATCCA-3’
SLC27A1	forward<br>reverse	5’-AGGTGGTTCAGTACATCGGG-3’<br>5’-AGAACTCCCCGATTTGGC-3’
ACBP	forward<br>reverse	5’-TGGCCACTACAAACAAGCAACT-3’<br>5’-TCCCGGGCCGTTCTG-3’
LIPE	forward<br>reverse	5’-GGAAGTGCTATCGTCTCTGG-3’<br>5’-GGCAGTCAGTGGCATCTC-3’
ACSL5	forward<br>reverse	5’-TGGCTTCACACAGGAGACATTG-3’<br>5’-GTTCTTTTTACGGTCGATGATCTTC-3’
FADS-2	forward<br>reverse	5’-CCCGGCACAACTTACACA-3’<br>5’-CCATGCTTGGCACATAGACACTT-3’
CPT-1	forward<br>reverse	5’TTTCCATTCCTTCCCATTCG-3’<br>5’GGGCTCGTGCGACATTTCT-3’
PLA2G6	forward<br>reverse	5’-TTTGTCACCCAAGAACTCTTAC-3’<br>5’-GGGAGGGAGGGTATGAGA-3’
PTGS1	forward<br>reverse	5’-ATGTATGAGTGTGGGATTTGA-3’<br>5’-TCCAAAATCCCTTGAAGTGGG-3’
SREBP2	forward<br>reverse	5’- TCCGCCTGTTCCGATGTAC-3’<br>5’- TGCACATTCAGCCAGGTTCA-3’
HMGCS	forward<br>reverse	5’-TTCACCATGCCTGGATCACTT-3’<br>5’-ATCTCAAGGGCAACAATTCCC-3’
HMGCR	forward<br>reverse	5’-GGATGACTCGTGGCCCAGT-3’<br>5’-TCGAGCCAGGCTTTCACTTC-3’
RPL32	forward<br>reverse	5’-GCACCAGTCAGACCGATATG-3’<br>5’-ACTGGGCAGCATGTGCTTTG-3’

Synthesis of TCS derivatives

NMR spectra were recorded at 30ºC on Varian INOVA 500 and 600 MHz NMR spectrometers. The latter spectrometer was equipped with a triple resonance cold probe. The ^{1}H and ^{13}C NMR chemical shifts were referenced to the solvent peak for $CDCl_3$ at δ_H 7.26 and δ_C 77.0. Coupling constants are given in Hz and chemical shifts are expressed as δ values in ppm. Column chromatography was undertaken on silica gel (Merck silica gel 60 (0.04-0.063 mm)) with HPLC grade solvents.

5-Chloro-2-(2,4-dichlorophenoxy)phenyl acetate (**3**). To a solution of **1** (15 mg, 0.05 mmol) in anhydrous pyridine (1 mL) was added acetic anhydride (10 μL, 0.10 mmol) and 4-dimethylaminopyridine (1 mg). The solution was stirred for 16 h, diluted with H_2O (10 mL), and the mixture was extracted with ethyl acetate (3 × 5 mL). The combined organic phase was washed with brine (5 mL), dried ($MgSO_4$), and concentrated in vacuo. The residue was

purified by silica gel column chromatography (5:1 hexane/ethyl acetate) to yield the title compound (6 mg, 42%) as a white solid. ^{1}H NMR (600 MHz, $CDCl_3$) δ 7.46 (d, J = 2.4 Hz, 1H), 7.22 – 7.13 (m, 3H), 6.87 (d, J = 8.8 Hz, 1H), 6.83 (d, J = 8.7 Hz, 1H), 2.22 (s, 3H) ppm; ^{13}C NMR (151 MHz, $CDCl_3$) δ 168.3, 151.0, 146.7, 141.7, 130.4, 129.6, 129.2, 128.1, 127.0, 126.0, 124.4, 120.5, 120.1, 20.4 ppm.

5-Chloro-2-(2,4-dichlorophenoxy)phenyl trifluoromethanesulfonate (**4**). To a solution of **1** (50 mg, 0.17 mmol) in anhydrous dichloromethane (1 mL) was added 2,4,6-collidine (91 μL, 0.69 mmol), and the solution was cooled to 0°C. Trifluoromethanesulfonic anhydride (35 μL, 0.20 mmol) was added, and the solution was allowed to warm to RT, and stirred for 16 h. The solution was diluted with H_2O (10 mL), and the mixture was extracted with ethyl acetate (3 × 5 mL). The combined organic phase was washed with brine (5 mL), dried ($MgSO_4$), and concentrated in vacuo. The residue was purified by silica gel column chromatography (10:1 hexane/ethyl acetate) to give the title compound (49 mg, 68%) as a white solid. ^{1}H NMR (600 MHz, $CDCl_3$) δ 7.40 (d, J = 2.4 Hz, 1H), 7.26 (d, J = 2.2 Hz, 1H), 7.20 – 7.10 (m, 3H), 6.91 (d, J = 8.7 Hz, 1H), 6.61 (d, J = 8.9 Hz, 1H) ppm; ^{13}C NMR (151 MHz, $CDCl_3$) δ 149.1, 147.5, 138.9, 131.3, 130.9, 129.4, 128.6, 128.5, 127.4, 123.6, 122.2, 118.6 (q, J = 320 Hz), 118.3 ppm.

5-Chloro-2-(2,4-dichlorophenoxy)benzonitrile (**5**). To a degassed solution of **4** (23 mg, 0.05 mmol) in anhydrous *N*,*N*-dimethylformamide (1 mL) was added $Zn(CN)_2$ (6.5 mg, 0.05 mmol) and $Pd(Ph_3P)_4$ (6 mg, 0.005 mmol). The reaction mixture was heated under microwave irradiation to 110°C for 1 h, then cooled to RT. The reaction mixture was diluted with H_2O (10 mL), and the mixture was extracted with ethyl acetate (3 × 5 mL). The combined organic phase was washed with brine (5 mL), dried ($MgSO_4$), and concentrated in vacuo. The residue was purified by silica gel column chromatography (20:1 hexane/ethyl acetate) to give the title compound (13 mg, 75%) as a white solid. ^{1}H NMR (600 MHz, $CDCl_3$) δ 7.65 (d, J = 2.5 Hz, 1H), 7.52 (d, J = 2.3 Hz, 1H), 7.42 (dd, J = 9.0, 2.4 Hz, 1H), 7.31 (dd, J = 8.7, 2.2 Hz, 1H), 7.09 (d, J = 8.7 Hz, 1H), 6.62 (d, J = 9.0 Hz, 1H). ^{13}C NMR (151 MHz, $CDCl_3$) δ 157.4, 148.8, 134.4, 133.4, 131.9, 131.1, 128.7, 128.4, 127.8, 123.3, 116.8, 114.2, 104.5 ppm.

(5-Chloro-2-(2,4-dichlorophenoxy)phenyl)methanol (**6**). To a solution of **5** (24 mg, 0.08 mmol) in anhydrous dichloromethane (1 mL) at -78°C was added diisobutylaluminium hydride solution (100 μL, 1M in dichloromethane) dropwise. The reaction mixture was stirred at this temperature for 1 h, then quenched by the addition of 1 M aqueous HCl (5 mL). The mixture was stirred at RT for 12 h, the extracted with dichloromethane (3 × 5 mL). The combined organic phase was washed with brine (5 mL), dried ($MgSO_4$), and concentrated in vacuo to give crude 5-chloro-2-(2,4-dichlorophenoxy)benzaldehyde as a white solid. The residue was suspended in anhydrous methanol (1 mL) and cooled to 0°C. Sodium borohydride (6 mg, 0.22 mmol) was added in one portion, and the reaction mixture was

allowed to warm to RT over 2 h. After this time, the reaction mixture was diluted with H_2O (10 mL), and the mixture was extracted with ethyl acetate (3 × 5 mL). The combined organic phase was washed with brine (5 mL), dried ($MgSO_4$), and concentrated in vacuo. The residue was purified by silica gel column chromatography (20:1 to 5:1 hexane/ethyl acetate) to give the title compound (15 mg, 63%) as a colourless oil. ^{1}H NMR (500 MHz, $CDCl_3$) δ 7.49 (d, J = 2.6 Hz, 1H), 7.48 (d, J = 2.5 Hz, 1H), 7.21 (dd, J = 8.7, 2.5 Hz, 1H), 7.18 (dd, J = 8.7, 2.6 Hz, 1H), 6.89 (d, J = 8.7 Hz, 1H), 6.63 (d, J = 8.7 Hz, 1H), 4.76 (s, 3H) ppm; ^{13}C NMR (151 MHz, $CDCl_3$) δ 152.5, 150.6, 132.8, 130.7, 129.9, 129.2, 129.0, 128.6, 128.2, 126.5, 121.2, 117.8, 60.3 ppm.

(**5-Chloro-2-(2,4-dichlorophenoxy)-phenoxy)acetic acid ethyl ester** (**7**). To a mixture of **1** (50 mg, 0.17 mmol), K_2CO_3 (100 mg, 0.72 mmol), sodium iodide (10 mg, 0.07 mmol) and Na_2HPO_4 (10 mg, 0.07 mmol) in anhydrous acetone (3 mL) was added ethyl chloroacetate (40 μL, 0.37 mmol), and the mixture was heated at reflux for 16 h. The reaction mixture was then cooled to RT, diluted with H_2O (20 mL), and extracted with ethyl acetate (3 × 10 mL). The combined organic phase was dried ($MgSO_4$) and concentrated in vacuo. The residue was purified by silica gel column chromatography (4:1 hexane/ethyl acetate) to give the title compound (60 mg, 94%) as a colourless oil. ^{1}H NMR (500 MHz, $CDCl_3$) δ 7.43 (d, J = 2.5 Hz, 1H), 7.12 (dd, J = 8.8, 2.5 Hz, 1H), 6.96 (dd, J = 8.5, 2.3 Hz, 1H), 6.93 (d, J = 2.3 Hz, 1H), 6.88 (d, J = 8.5 Hz, 1H), 6.77 (d, J = 8.8 Hz, 1H), 4.63 (s, 2H), 4.23 (q, J = 7.2 Hz, 2H), 1.27 (d, J = 7.2 Hz, 3H) ppm; 168.0, 151.8, 149.7, 143.7, 130.2, 130.1, 128.4, 127.8, 124.8, 122.6, 121.7, 118.8, 116.0, 66.4, 61.5, 14.1 ppm.

(5-Chloro-2-(2,4-dichlorophenoxy)-phenoxy)acetic acid (**8**). A solution of **7** (48 mg, 0.13 mmol) in 2 M NaOH solution (1 mL) was heated to 90°C for 2 h. The reaction mixture was cooled to 0°C, and the pH of the mixture was adjusted to 2 by the addition of 1 M HCl solution. The aqueous phase was extracted with ethyl acetate (3 × 10 mL) and the combined organic phase was concentrated in vacuo. The residue was purified by silica gel column chromatography (1:1 hexane/ethyl acetate) to give the title compound (23 mg, 52%) as a colourless oil. ^{1}H NMR (500 MHz, $CDCl_3$) δ 7.46 (s, 1H), 7.16 (d, J = 8.9 Hz, 1H), 7.01 (m, 2H), 6.88 (d, J = 8.9 Hz, 1H), 6.80 (d, J = 8.8 Hz, 1H), 4.73 (s, 1H) ppm; ^{13}C NMR (125 MHz, $CDCl_3$) δ 172.7, 151.5, 149.2, 144.1, 130.4, 130.1, 128.8, 127.9, 125.2, 123.1, 121.3, 119.2, 116.5, 66.1 ppm.

The synthesis flow chart and structures are shown below. For comparison the commercially available methyl triclosan (TCSm) [5-Chloro-2-(2,4-dichlorophenoxy)anisole] (**2**) (Sigma) is displayed as well.

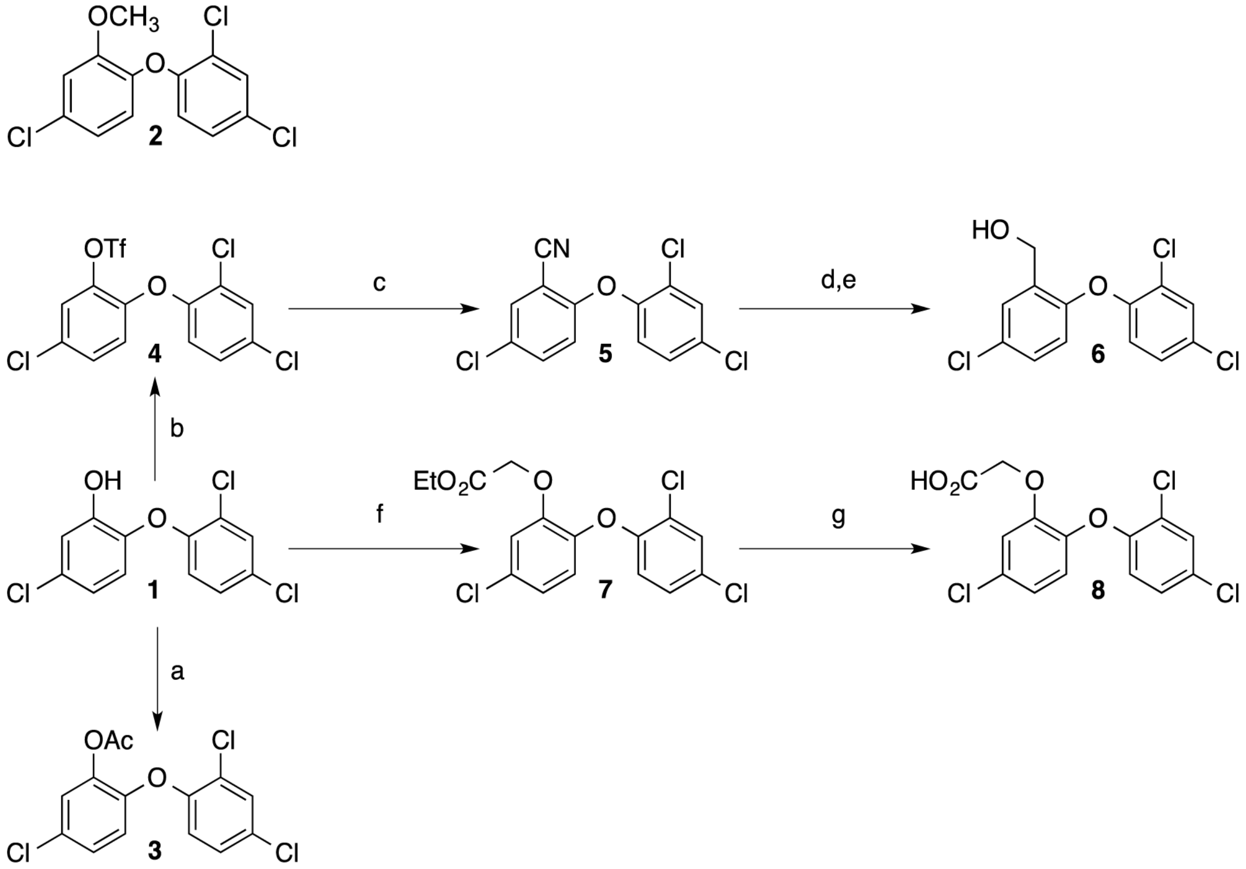

Supplement: Supplementary file 1 [file oncotarget-05-9362-s001.pdf]
